# COVID-19 Vaccination Site Accessibility, United States, December 11, 2020–March 29, 2022

**DOI:** 10.3201/eid3005.230357

**Published:** 2024-05

**Authors:** Randy Yee, David Carranza, Christine Kim, James Phillip Trinidad, James L. Tobias, Roma Bhatkoti, Sachiko Kuwabara

**Affiliations:** Centers for Disease Control and Prevention, Atlanta, Georgia, USA (R. Yee, D. Carranza, C. Kim, J.L. Tobias, R. Bhakoti, S. Kuwabara);; US Department of Health and Human Services Coordination Operations and Response Element, Washington, DC (J.P. Trinidad)

**Keywords:** COVID-19, 2019 novel coronavirus disease, coronavirus disease, severe acute respiratory syndrome coronavirus 2, SARS-CoV-2, viruses, respiratory infections, zoonoses, accessibility, GIS analysis, vaccination sites, vaccine, social vulnerability index, United States

## Abstract

During December 11, 2020–March 29, 2022, the US government delivered ≈700 million doses of COVID-19 vaccine to vaccination sites, resulting in vaccination of ≈75% of US adults during that period. We evaluated accessibility of vaccination sites. Sites were accessible by walking within 15 minutes by 46.6% of persons, 30 minutes by 74.8%, 45 minutes by 82.8%, and 60 minutes by 86.7%. When limited to populations in counties with high social vulnerability, accessibility by walking was 55.3%, 81.1%, 86.7%, and 89.4%, respectively. By driving, lowest accessibility was 96.5% at 15 minutes. For urban/rural categories, the 15-minute walking accessibility between noncore and large central metropolitan areas ranged from 27.2% to 65.1%; driving accessibility was 79.9% to 99.5%. By 30 minutes driving accessibility for all urban/rural categories was >95.9%. Walking time variations across jurisdictions and between urban/rural areas indicate that potential gains could have been made by improving walkability or making transportation more readily available.

On December 11, 2020, the US Food and Drug Administration (FDA) authorized the first COVID-19 vaccine for emergency use for persons >16 years of age (*1*). Although during the initial period of limited supply the vaccine was allocated to at-risk older adults and certain essential workers, by the end of spring 2021 vaccine eligibility gradually expanded to all adults (*2*). On September 22, 2021, the Pfizer-BioNTech (https://www.pfizer.com) COVID-19 vaccine emergency use authorization was amended to allow a single booster dose for elderly adults and adults with underlying medical conditions. By November 19, 2021, a booster dose was recommended for all adults (*1*), and on March 29, 2022, emergency use authorization allowed for a second booster dose (*3*). By March 29, 2022, the percentage of adults who had completed the primary COVID-19 vaccination series was 75.4%, and 48.2% had received a booster dose (*4*).

A major structural barrier of the US COVID-19 vaccination campaign is vaccine accessibility. The Assistant Secretary for Planning and Evaluation, Office of Health Policy, found that major barriers to vaccine coverage were transportation-related costs, opportunity costs, and inadequate functional proximity to the vaccines (*5*). The US Centers for Disease Control and Prevention (CDC) has provided recommendations to jurisdictions with regard to the planning of convenient COVID-19 vaccination sites, including use of gap analysis to spatially assess optimal locations for additional sites, especially those with populations of homebound persons or persons living in remote places (*6*,*7*).

Another major theme of the vaccination campaign has been equity of access. The CDC COVID-19 Response Health Equity Strategy has outlined tangible activities for ensuring equity in the nationwide distribution and administration of COVID-19 vaccines through data-driven approaches such as expanding vaccine data collection, reporting, and analyses (*8*). Organizations such as the Office for Civil Rights also have highlighted accessibility challenges and the need for federally assisted healthcare providers to ensure fair and equitable access to vaccines and boosters, citing laws such as Title VI of the Civil Rights Act of 1964 and Section 1557 of the Affordable Care Act (*9*). The White House has repeatedly confirmed its commitment to ensuring equitable access to COVID-19 vaccines (*10*–*12*).

Geographic information systems have proven valuable for evaluating functional proximity of populations to vaccination sites; geospatial analyses use dynamic travel time estimation methods for vaccine modeling and planning increasing during the COVID-19 pandemic (*13*–*21*). For example, Whitehead et al. calculated travel times between Aotearoa, the New Zealand census-derived Statistical Area 1 level, to all potential vaccine delivery sites with a road network and origin/destination analysis (J. Whitehead et al., unpub. data, https://www.medrxiv.org/content/10.1101/2021.08.26.21262647v1). In a nationwide study in England, Duffy et al. measured accessibility from statistical and administrative neighborhood centroids to COVID-19 vaccination sites by using the closest travel time routes and the most likely transport mode in each area: by car, by public transport, or on foot (*20*). Another study conducted in Kenya used a cost–distance algorithm based on walking and motorized modes to generate a gridded travel time surface at the 1 × 1–km spatial resolution level together with a population density layer from WorldPop (https://www.worldpop.org) to determine accessibility of population to vaccination centers (*21*). A Bayesian conditional autoregressive model was then used to identify inequalities in accessibility and to predict vaccination coverage rates (*21*).

Knowledge of the functional proximity to vaccine sites for different populations is essential for effective planning and for ensuring equity of health resource access in public health emergencies. With our retrospective study, we evaluated accessibility of COVID-19 vaccine sites nationally by walking and driving travel times at the jurisdiction, community social vulnerability, and urban/rural levels. 

The study was approved by the CDC National Center for Health Statistics. CDC investigators did not interact with human subjects or have access to identifiable data or specimens.

## Methods

To obtain a list of active provider sites, we queried the official platform for US COVID-19 vaccine distribution, the Tiberius system (https://www.cdc.gov/vaccines/programs/tiberius/index.html). We defined an active provider site as any provider site from December 11, 2020 (with FDA authorization for emergency use of the first COVID-19 vaccine, Pfizer-BioNTech) through March 29, 2022 (with FDA authorization of a second booster dose for older and immunocompromised persons) that at any point reported any inventory in the preceding 7 days, received a shipment in the preceding 28 days, or administered >1 dose of adult COVID-19 vaccine during the preceding 28 days (*1*,*3*). Sites must have been a provider of one of the approved or authorized adult COVID-19 vaccines in the United States during the study period: Pfizer-BioNTech, Moderna (https://www.modernatx.com), and Janssen (https://www.jnj.com). To avoid potentially overestimating provider site accessibility for the general population, we excluded sites serving institutionalized or long-term resident populations (e.g., prisons, detention facilities, and nursing homes) or military personnel (e.g., Department of Defense provider sites).

We grouped provider sites into 1 of 5 self-reported categories: community health, hospital, medical practice, pharmacy, and unknown/other (Appendix Figure 1). Community health providers were reported as public health, tribal health, or commercial vaccination service sites. The hospital category included only those reporting as hospital providers. Medical practice providers were reported as doctor’s offices or practices, health centers, urgent care, or Indian Health Service. Pharmacy sites were reported as retail pharmacy or other pharmacy sites. Sites categorized as unknown/other were either reported as home health, other, or no selection.

**Figure 1:**
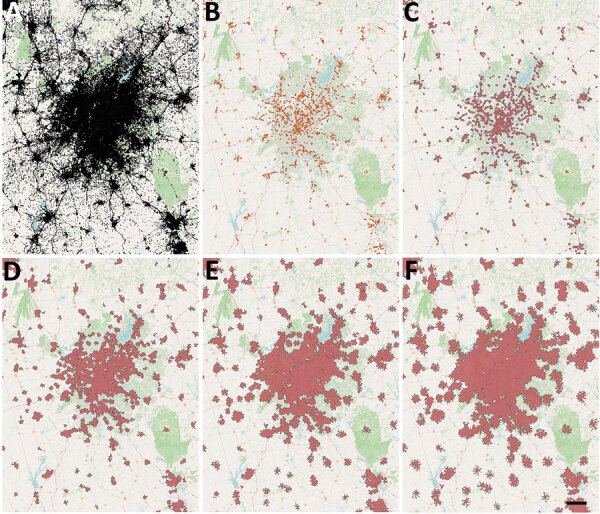
COVID-19 vaccination site accessibility, Atlanta metropolitan area, Georgia, USA, December 11, 2020–March 29, 2022. A) Adult population density; B) COVID-19 providers; C) COVID-19 vaccination site accessible by 15-minute walk; D) 30-minute walk; E) 45-minute walk; F) 60-minute walk. Scale bar indicates 20 km.

To calculate isochrones (areas from which a site can be reached within a specific travel time) for each provider site we used OpenRouteService (https://openrouteservice.org), an open-source global spatial routing service. We obtained the latest OpenStreetMaps road data for the United States (https://planet.openstreetmap.org) from Geofabrik (http://www.geofabrik.de). We deployed OpenRouteService locally on Docker, a containerization platform for creating and running applications, and accessed it through Quantum GIS (http://www.qgis.org) via the OpenRouteService Tools plugin (*22**,**23*). We generated isochrones around each site for walking and driving with the time thresholds of 15, 30, 45, and 60 minutes. We calculated walking and driving travel speeds by using the OpenRouteService algorithm, which is described in their documentation. For the underlying US adult population raster layer, we obtained 2019 US population estimates for female/male >15-year age bands constrained to built settlements from WorldPop and aggregated the data in Python (*24*,*25*). We then calculated site accessibility by overlaying the isochrone contours on top of the population layer and using zonal statistics in Quantum GIS to determine the population residing within the isochrones (Figure 1).

We evaluated accessibilities for US adults living at different community social vulnerability levels and urbanicity by using the CDC/Agency for Toxic Substances and Disease Registry Social Vulnerability Index (SVI), a composite index based on 15 US Census variables grouped by socioeconomic status, household composition and disability, minority status and language, and housing and transportation (*26*). The most recent SVI was released in 2018, and the range was 0–1, with 1 representing the highest vulnerability (*26*). High SVI was defined as an overall SVI >0.5 and was used as a filter for the population residing in those areas.

The National Center for Health Statistics Urban-Rural Classification Scheme for 2013 classified counties into 1 of 6 categories: large central metropolitan, large fringe metropolitan, medium metropolitan, small metropolitan, micropolitan, and noncore (core urban population <10,000) (*27*). We aggregated the adult population for each of these urban/rural areas and calculated accessibilities by using the same isochrones generated previously.

## Results

The total number of active provider sites for the study period was 131,951. There were 57,064 pharmacy sites, 35,728 medical practice sites, 10,606 community health sites, 5,222 hospitals, and 23,331 unknown or other provider site type (Appendix Figure 2). 

**Figure 2:**
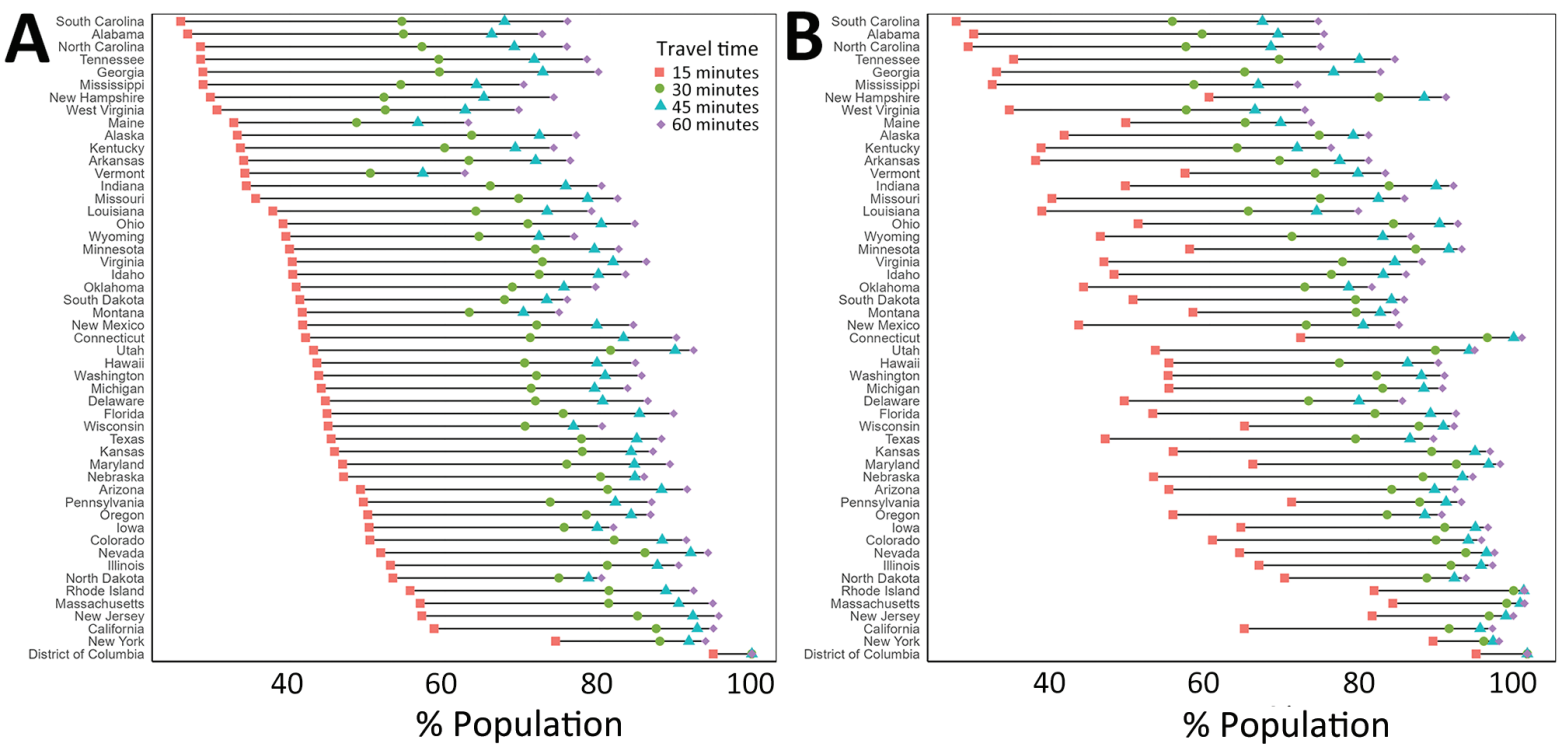
Adult COVID-19 vaccination site accessibility according to walking time in 15-minute intervals, by state, United States, December 11, 2020–March 29, 2022. A) Overall accessibility; B) accessibility for areas with high scores on the Centers for Disease Control and Prevention/Agency for Toxic Substances and Disease Registry Social Vulnerability Index.

**Figure 3:**
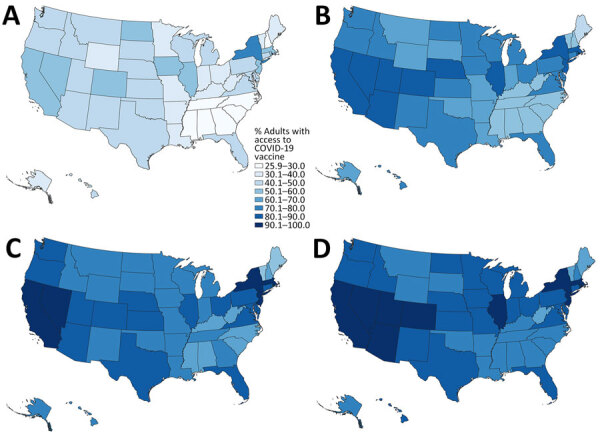
Walking accessibility for COVID-19 vaccination sites, by state, United States, December 11, 2020–March 29, 2022: A) 15 minutes; B) 30 minutes; C) 45 minutes; D) 60 minutes.

**Figure 4:**
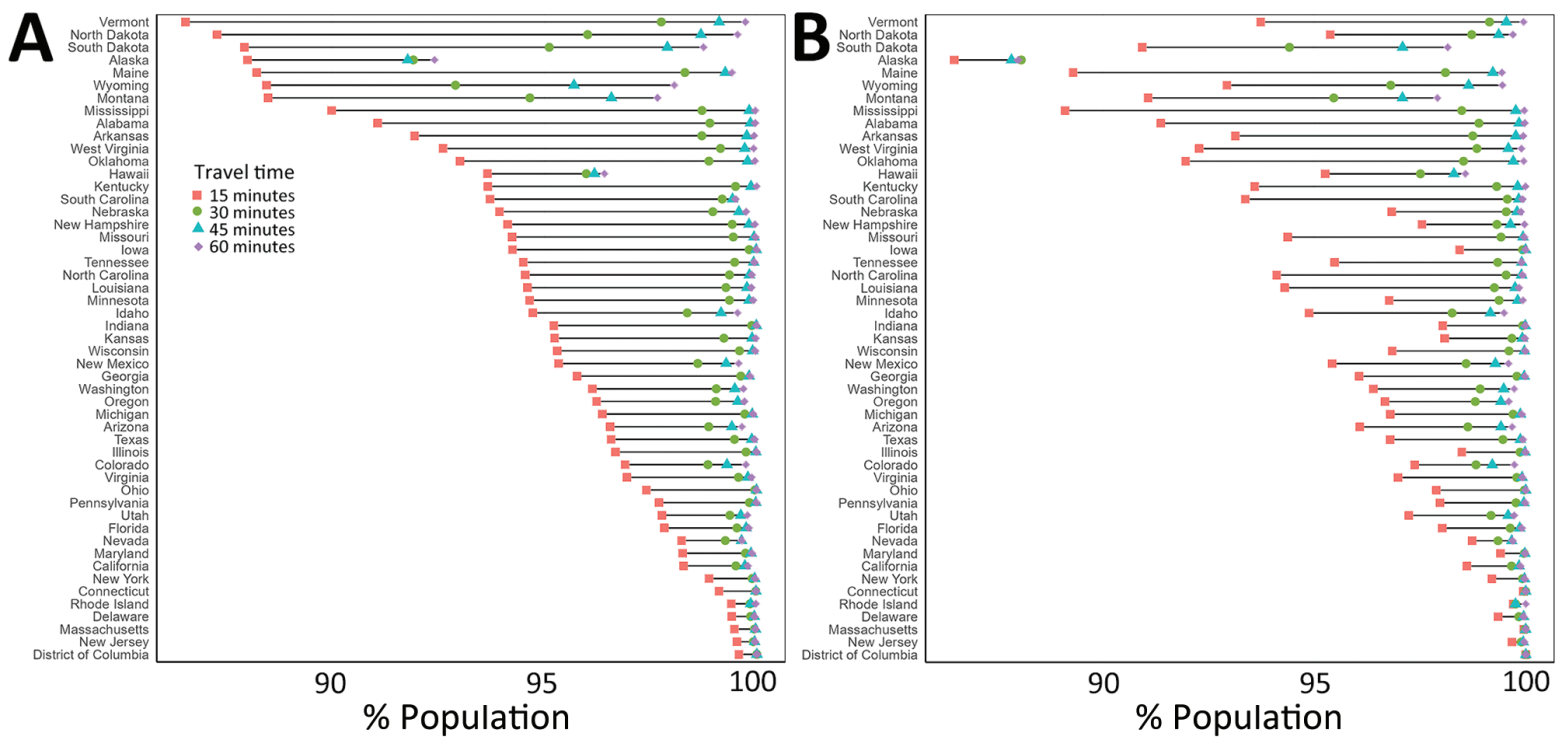
Adult COVID-19 vaccination site accessibility according to driving time in 15-minute intervals, by state, United States, December 11, 2020–March 29, 2022. A) Overall accessibility; B) accessibility for areas with high scores on the Centers for Disease Control and Prevention/Agency for Toxic Substances and Disease Registry Social Vulnerability Index.

**Figure 5:**
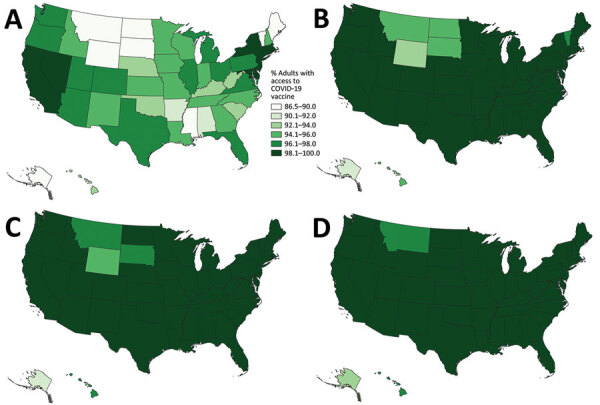
Driving accessibility for COVID-19 vaccination site, by state, United States, December 11, 2020–March 29, 2022. A) 15 minutes; B) 30 minutes; C) 45 minutes; D) 60 minutes.

National walking accessibilities (the proportion of the population within the stated travel time areas) were found to be 46.6% within 15 minutes, 74.8% within 30, 82.8% within 45 minutes, and 86.7% within 60 minutes. The national driving accessibilities for those times were 96.5%, 99.4%, 99.7%, and 99.8%, respectively (Figures 2, 3, 4, 5).

When we limited the population to persons residing in high SVI areas, sites were accessible within a 15-minute walk for 55.3% of the population, 30-minute walk for 81.1%, 45-minute walk for 86.7%, and 60-minute walk for 89.4%; accessibilities for those same times by driving were 97.0%, 99.5%, 99.8%, and 99.9%, respectively (Figures 2, 4). Overall, accessibility was greater in high-SVI areas than in the entire area.

**Figure 6:**
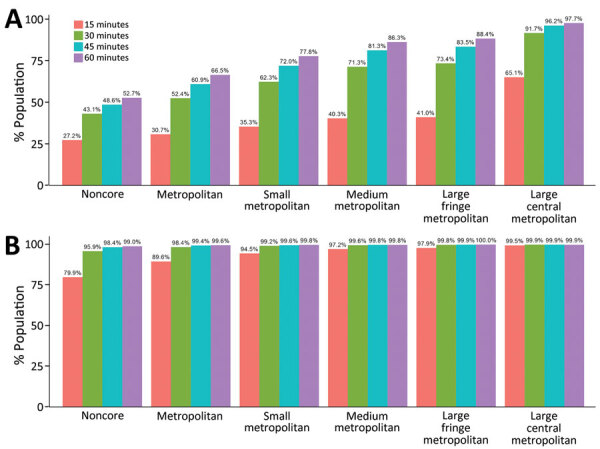
Adult COVID-19 vaccination site accessibility by 2013 Centers for Disease Control and Prevention Urban-Rural Classification, United States, December 11, 2020–March 29, 2022. A) Walking accessibility; B) driving accessibility.

Accessibility tended to improve with increasing urbanicity of the location of provider sites. The noncore walking accessibilities ranged from 15 minutes (27.2%) to 60 minutes (52.7%), and accessibilities for large central metropolitan areas ranged from 65.1% to 97.7%. Driving accessibility ranges were 79.9%–99.0% for noncore and 99.5%–99.9% for large central metropolitan areas (Figure 6).

## Discussion

With this study, we estimated a range of accessibilities to COVID-19 vaccination sites for US adults: 46.6% of US adults were within a 15-minute walk to a COVID-19 vaccine provider site, which increased to 86.7% for a 60-minute walk. Accessibility increased further, to 96.5%, for a 15-minute drive. Although access tended to be greater than or equal for high-SVI areas compared with the entire jurisdiction, jurisdictional and urban/rural variations suggest potential inequalities with regard to access. Although we do not attempt to say what the accessibility target should be for each area, future public health campaigns should factor these perspectives into planning.

Given that driving has the potential to greatly increase accessibility, making motorized transportation more readily available to areas in which walking times are high should be considered. Despite rural counties having more households with cars, large numbers of zero-car ownership households depend on underfunded public transportation systems (*28*,*29*). Vaccine transportation programs such as MyTurn (https://myturn.ca.gov) and those offered by rideshare services such as Uber and Lyft were initiated as part of the response, but whether they are situated for maximum effect could be a topic for another study (*30*,*31*). Conversely, another perspective worth investigating is strategic placement of vaccination sites, such as mobile clinics or temporary vaccination sites for walking.

Our analysis considers only 2 travel modalities: walking and driving. We acknowledge that those modalities might not be suitable or preferred by everyone. For example, the average travel time for a person requiring use of a wheelchair may be greater than the walking speeds calculated by using the OpenRouteService algorithm. Public transportation, another common mode of travel, can vary according to traffic conditions and whether dedicated travel lanes are available. Nonetheless, walking and driving should give a reasonable range with regard to accessibility of the vaccine.

Among potential limitations of the provider list, not all vaccine provider sites are equally accessible to the public. Provider sites may have policies that restricted access to specific groups of persons or hours of operation that may have excluded persons. Some sites may not be accessible at all because our selection criteria capture sites that are not intended for vaccine administration, such as distribution hubs. Our study assessed only accessibility of static sites and did not consider mobile vaccination clinics or services that provide in-home vaccination. Our study reports the cumulative list of active provider sites, including sites that were not active during the entire period, such as mass vaccination sites operational only during the early months of the vaccination campaign. Site inactivation is a relevant consideration; a recent study has shown how closures can result in heavy losses of access resulting from increased driving travel times in the more rural parts of the country (*32*). Furthermore, our analysis did not account for characteristics of the provider vaccine supply, such as availability of specific vaccines or vaccine stockouts during the study period. The criteria for being an active provider depend on accurate data. The Tiberius platform data are frequently updated, and provider data can be updated days or even weeks later. Although the provider list was pulled well after the close of the study period, those delays could still shift the number of providers flagged as active, depending on the time of data export.

Another limitation is that our analysis evaluated only proximity to a vaccine provider site via established road networks. OpenStreetMaps is an open-sourced project, and although the project is regularly updated by independent contributors, there may be network gaps such as informal roads or hidden back roads. In addition, connecting isochrone endpoints on road networks can produce minor differences in the polygon contours, depending on the method used. Numerous resources compare results between different routing services (*33*).

One more limitation was timeliness of the geospatial data sources. Worldpop estimates were produced in 2019; the CDC/Agency for Toxic Substances and Disease Registry SVI in 2018; and the National Center for Health Statistics Urban/Rural Classification Scheme in 2013 (*24*,*26*,*27*). Furthermore, we used population estimates for persons >15 years of age to approximate those eligible for the vaccine within the study period as well as those able to drive. Those estimates may overestimate the driving accessibility for some jurisdictions because driving age does not start at age 15 for all states. Because the estimates were produced in 2019, at the time of our analysis, the entire estimated population would be a few years older (more persons >15 years of age), better estimating the driving population during the study. Researchers using our methods should consider the timeliness of their data sources and should either contact the data providers directly or use estimation methods if data sources are out of date. Despite those limitations, we assert the value of our methods and results for vaccine planning purposes.

Our article addresses only the issue of physical access, but accessibility itself is a multifaceted concept. For example, Levesque et al. developed a framework with 5 dimensions of accessibility: approachability, acceptability, availability and accommodation, affordability, and appropriateness (*34*). Although we have explored some aspects of availability and accommodation, future work could focus on evaluating accessibility along these other dimensions.

In conclusion, the initial US COVID-19 vaccination campaign was a monumental logistical undertaking resulting in deliveries of ≈700 million doses to fully vaccinate ≈75% of US adults by the end of the study period. Despite that achievement, our analysis has identified potential areas for improvement at the national, jurisdiction, and urban/rural levels. Our findings highlight the value of evaluating accessibility at different levels for vaccine planning.

Appendix Additional information for study of COVID-19 vaccination site accessibility, United States, December 11, 2020–March 29, 2022.
