# Supplementary material for: COVID-19 Vaccination Site Accessibility, United States, December 11, 2020–March 29, 2022
Source: Emerg Infect Dis. 2024 May;30(5):947–55. doi: 10.3201/eid3005.230357 (PMC11060460; doi:10.3201/eid3005.230357)
Supplement: Appendix — Additional information for study of COVID-19 vaccination site accessibility, United States, December 11, 2020–March 29, 2022. [file 23-0357-Techapp-s1.pdf]

Article DOI: https://doi.org/10.3201/eid3005.230357

EID cannot ensure accessibility for supplementary materials supplied by authors. Readers who have difficulty accessing supplementary content should contact the authors for assistance.

COVID-19 Vaccination Site Accessibility, United States, December 11, 2020–March 29, 2022

Appendix

Appendix Table 1. Estimated accessibility of adult COVID-19 vaccine by 2013 CDC Urban-Rural Classification

Modality	Urban-Rural Classification	15 min.	30 min.	45 min.	60 min.
Walking	Large Central Metro	65.1%	91.7%	96.2%	97.7%
	Large Fringe Metro	41.0%	73.4%	83.5%	88.4%
	Medium Metro	40.3%	71.3%	81.3%	86.3%
	Small Metro	35.3%	62.3%	72.0%	77.8%
	Micropolitan	30.7%	52.4%	60.9%	66.5%
	Non-core	27.2%	43.1%	48.6%	52.7%
Driving	Large Central Metro	99.5%	99.9%	99.9%	99.9%
	Large Fringe Metro	97.9%	99.8%	99.9%	100.0%
	Medium Metro	97.2%	99.6%	99.8%	99.8%
	Small Metro	94.5%	99.2%	99.6%	99.8%
	Micropolitan	89.6%	98.4%	99.4%	99.6%
	Non-core	79.9%	95.9%	98.4%	99.0%

Appendix Table 2. Estimated accessibility of adult COVID-19 vaccine by jurisdiction

	Walking				Driving			
State	15 Min.	30 Min.	45 Min.	60 Min.	15 Min.	30 Min.	45 Min.	60 Min.
Alabama	26.8%	54.6%	66.0%	72.6%	91.1%	98.9%	99.8%	100.0%
Alaska	33.2%	63.4%	72.2%	77.0%	88.0%	91.9%	91.8%	92.4%
Arizona	49.1%	81.0%	88.0%	91.3%	96.5%	98.9%	99.4%	99.6%
Arkansas	34.0%	63.1%	71.7%	76.2%	91.9%	98.7%	99.8%	99.9%
California	58.6%	87.3%	92.6%	94.7%	98.3%	99.5%	99.7%	99.8%
Colorado	50.3%	81.9%	88.1%	91.2%	96.9%	98.8%	99.3%	99.7%
Connecticut	42.0%	71.0%	83.1%	89.9%	99.1%	100.0%	100.0%	100.0%
Delaware	44.5%	71.7%	80.4%	86.2%	99.4%	99.9%	99.9%	99.9%
District of Columbia	94.7%	99.6%	99.6%	99.6%	99.6%	100.0%	100.0%	100.0%
Florida	44.8%	75.3%	85.1%	89.5%	97.8%	99.5%	99.7%	99.8%
Georgia	28.7%	59.3%	72.6%	79.8%	95.8%	99.6%	99.8%	99.8%
Hawaii	43.4%	70.3%	79.6%	84.6%	93.7%	96.0%	96.2%	96.4%
Idaho	40.3%	72.2%	79.8%	83.3%	94.7%	98.4%	99.2%	99.5%
Illinois	52.9%	81.0%	87.4%	90.2%	96.7%	99.7%	100.0%	100.0%
Indiana	34.3%	65.8%	75.6%	80.2%	95.2%	99.9%	100.0%	100.0%
Iowa	50.2%	75.4%	79.7%	81.8%	94.2%	99.8%	100.0%	100.0%
Kansas	45.7%	77.7%	84.0%	86.9%	95.2%	99.2%	99.9%	100.0%
Kentucky	33.6%	59.9%	69.1%	74.1%	93.7%	99.5%	99.9%	100.0%
Louisiana	37.8%	64.0%	73.2%	78.9%	94.6%	99.3%	99.8%	99.9%
Maine	32.7%	48.6%	56.5%	63.0%	88.2%	98.3%	99.3%	99.4%
Maryland	46.8%	75.7%	84.5%	89.1%	98.3%	99.7%	99.9%	99.9%
Massachusetts	56.8%	81.1%	90.2%	94.6%	99.5%	99.9%	100.0%	100.0%
Michigan	44.0%	71.1%	79.3%	83.6%	96.4%	99.7%	99.9%	99.9%
Minnesota	39.9%	71.7%	79.3%	82.5%	94.6%	99.4%	99.8%	99.9%
Mississippi	28.8%	54.3%	64.1%	70.2%	90.0%	98.7%	99.8%	100.0%
Missouri	35.5%	69.5%	78.4%	82.3%	94.2%	99.4%	99.9%	100.0%
Montana	41.6%	63.1%	70.1%	74.7%	88.5%	94.7%	96.6%	97.7%
Nebraska	46.9%	80.1%	84.5%	85.7%	93.9%	99.0%	99.6%	99.8%

State	Walking				Driving			
	15 Min.	30 Min.	45 Min.	60 Min.	15 Min.	30 Min.	45 Min.	60 Min.
Nevada	51.7%	85.8%	91.7%	94.0%	98.2%	99.3%	99.6%	99.6%
New Hampshire	29.7%	52.1%	65.0%	74.1%	94.1%	99.4%	99.8%	100.0%
New Jersey	57.0%	84.9%	92.0%	95.4%	99.5%	99.9%	99.9%	100.0%
New Mexico	41.6%	71.9%	79.6%	84.3%	95.3%	98.6%	99.3%	99.6%
New York	74.3%	87.8%	91.5%	93.7%	98.9%	99.9%	100.0%	100.0%
North Carolina	28.4%	57.0%	69.0%	75.7%	94.5%	99.4%	99.8%	99.9%
North Dakota	53.3%	74.7%	78.6%	80.2%	87.3%	96.0%	98.7%	99.5%
Ohio	39.1%	70.7%	80.2%	84.5%	97.4%	100.0%	100.0%	100.0%
Oklahoma	40.8%	68.7%	75.4%	79.5%	93.0%	98.9%	99.8%	100.0%
Oregon	50.0%	78.3%	84.1%	86.6%	96.2%	99.0%	99.6%	99.7%
Pennsylvania	49.4%	73.6%	82.0%	86.7%	97.7%	99.8%	100.0%	100.0%
Rhode Island	55.5%	81.2%	88.5%	92.1%	99.4%	99.8%	99.9%	100.0%
South Carolina	25.9%	54.4%	67.7%	75.8%	93.7%	99.2%	99.4%	99.5%
South Dakota	41.3%	67.7%	73.2%	75.8%	87.9%	95.1%	97.9%	98.7%
Tennessee	28.4%	59.2%	71.5%	78.4%	94.5%	99.5%	99.9%	99.9%
Texas	45.3%	77.6%	84.8%	88.0%	96.6%	99.5%	99.9%	100.0%
Utah	43.0%	81.4%	89.7%	92.1%	97.8%	99.4%	99.6%	99.8%
Vermont	34.1%	50.4%	57.1%	62.6%	86.5%	97.7%	99.1%	99.7%
Virginia	40.3%	72.6%	81.7%	86.0%	96.9%	99.6%	99.8%	99.9%
Washington	43.7%	71.8%	80.7%	85.4%	96.1%	99.0%	99.5%	99.7%
West Virginia	30.6%	52.3%	62.6%	69.5%	92.6%	99.1%	99.7%	99.9%
Wisconsin	44.9%	70.3%	76.6%	80.3%	95.3%	99.6%	99.9%	100.0%
Wyoming	39.4%	64.4%	72.2%	76.7%	88.4%	92.9%	95.7%	98.1%
National	46.6%	74.8%	82.8%	86.7%	96.5%	99.4%	99.7%	99.8%

Appendix Table 3. Estimated high SVI accessibility (SVI > 0.5) of adult COVID-19 vaccine by jurisdiction

State	Walking				Driving			
	15 Min.	30 Min.	45 Min.	60 Min.	15 Min.	30 Min.	45 Min.	60 Min.
Alabama	30.3%	59.0%	68.6%	74.4%	91.4%	98.9%	99.8%	100.0%
Alaska	41.7%	73.8%	78.1%	80.0%	86.5%	88.1%	87.8%	88.0%
Arizona	54.9%	82.9%	88.3%	90.9%	96.1%	98.6%	99.4%	99.7%
Arkansas	38.1%	68.8%	76.4%	80.0%	93.1%	98.7%	99.8%	99.9%
California	64.4%	90.1%	94.0%	95.6%	98.6%	99.7%	99.8%	99.9%
Colorado	60.4%	88.5%	92.5%	94.2%	97.4%	98.8%	99.2%	99.7%
Connecticut	71.4%	94.9%	98.3%	99.3%	99.9%	100.0%	100.0%	100.0%
Delaware	49.3%	72.5%	78.8%	84.2%	99.3%	99.8%	99.9%	100.0%
District of Columbia	93.5%	100.0%	100.0%	100.0%	100.0%	100.0%	100.0%	100.0%
Florida	52.8%	80.8%	87.8%	91.0%	98.0%	99.6%	99.9%	99.9%
Georgia	33.2%	64.4%	75.6%	81.5%	96.1%	99.8%	100.0%	100.0%
Hawaii	54.9%	76.3%	84.9%	88.8%	95.3%	97.5%	98.3%	98.6%
Idaho	48.0%	75.3%	81.9%	84.7%	94.9%	98.3%	99.2%	99.5%
Illinois	66.2%	90.4%	94.2%	95.6%	98.5%	99.9%	100.0%	100.0%
Indiana	49.4%	82.6%	88.5%	90.7%	98.0%	99.9%	100.0%	100.0%
Iowa	63.9%	89.6%	93.4%	95.0%	98.4%	99.9%	100.0%	100.0%
Kansas	55.4%	87.9%	93.4%	95.3%	98.1%	99.7%	99.9%	100.0%
Kentucky	38.8%	63.5%	71.0%	75.3%	93.6%	99.3%	99.8%	100.0%
Louisiana	38.9%	64.9%	73.5%	78.7%	94.3%	99.3%	99.7%	99.8%
Maine	49.4%	64.5%	68.9%	72.8%	89.3%	98.1%	99.2%	99.4%
Maryland	65.4%	91.0%	95.1%	96.6%	99.4%	100.0%	100.0%	100.0%
Massachusetts	83.0%	97.4%	99.1%	99.6%	100.0%	100.0%	100.0%	100.0%
Michigan	54.9%	81.8%	87.0%	89.3%	96.8%	99.7%	99.9%	99.9%
Minnesota	57.5%	85.9%	90.1%	91.7%	96.8%	99.4%	99.8%	99.9%
Mississippi	32.6%	58.0%	66.1%	71.1%	89.1%	98.5%	99.8%	100.0%
Missouri	40.2%	73.9%	81.2%	84.5%	94.4%	99.4%	99.9%	100.0%
Montana	57.9%	78.4%	81.5%	83.4%	91.1%	95.5%	97.1%	97.9%
Nebraska	52.9%	86.8%	91.8%	93.1%	96.8%	99.5%	99.8%	99.9%
Nevada	63.8%	92.2%	94.8%	95.9%	98.7%	99.3%	99.7%	99.7%
New Hampshire	59.9%	81.3%	87.0%	89.8%	97.5%	99.3%	99.6%	100.0%
New Jersey	80.4%	95.2%	97.3%	98.2%	99.7%	99.9%	99.9%	100.0%
New Mexico	43.5%	72.2%	79.3%	83.8%	95.4%	98.6%	99.3%	99.6%
New York	88.1%	94.5%	95.7%	96.4%	99.2%	99.9%	100.0%	100.0%
North Carolina	29.6%	57.0%	67.7%	73.9%	94.1%	99.5%	99.9%	99.9%
North Dakota	69.4%	87.3%	90.8%	92.3%	95.4%	98.7%	99.4%	99.7%
Ohio	51.0%	83.1%	88.9%	91.2%	97.9%	100.0%	100.0%	100.0%
Oklahoma	44.1%	72.0%	77.5%	80.4%	92.0%	98.5%	99.7%	100.0%

	Walking				Driving			
State	15 Min.	30 Min.	45 Min.	60 Min.	15 Min.	30 Min.	45 Min.	60 Min.
Oregon	55.4%	82.3%	87.1%	89.2%	96.7%	98.8%	99.4%	99.6%
Pennsylvania	70.3%	86.4%	89.8%	91.7%	98.0%	99.8%	100.0%	100.0%
Rhode Island	80.7%	98.2%	99.5%	99.6%	99.7%	99.8%	99.8%	100.0%
South Carolina	28.1%	55.3%	66.6%	73.7%	93.4%	99.6%	99.8%	99.9%
South Dakota	50.4%	78.4%	82.9%	84.5%	90.9%	94.4%	97.1%	98.2%
Tennessee	35.3%	68.7%	78.9%	83.3%	95.5%	99.3%	99.9%	99.9%
Texas	46.9%	78.4%	85.2%	88.2%	96.8%	99.5%	99.9%	99.9%
Utah	53.2%	88.4%	92.7%	93.4%	97.2%	99.2%	99.6%	99.7%
Vermont	56.9%	73.3%	78.7%	82.1%	93.7%	99.1%	99.5%	99.9%
Virginia	46.7%	76.7%	83.3%	86.7%	97.0%	99.8%	99.9%	99.9%
Washington	54.8%	81.0%	86.7%	89.6%	96.4%	98.9%	99.5%	99.7%
West Virginia	34.8%	57.1%	65.7%	72.0%	92.3%	98.8%	99.6%	99.9%
Wisconsin	64.4%	86.3%	89.4%	90.8%	96.8%	99.6%	100.0%	100.0%
Wyoming	46.2%	70.4%	81.8%	85.3%	92.9%	96.8%	98.7%	99.4%
Alabama	30.3%	59.0%	68.6%	74.4%	91.4%	98.9%	99.8%	100.0%
**National**	**55.3%**	**81.1%**	**86.7%**	**89.4%**	**97.0%**	**99.5%**	**99.8%**	**99.9%**

Community Health

- Commercial vaccination service provider
- Tribal health
- Public health provider - public health clinic
- Public health provider - Federally Qualified Health Center
- Public health provider - Rural Health Clinic

Hospital

- Hospital

Medical Practice

- Health center - migrant or refugee
- Health center - occupational
- Health cetner - STD/HIV clinic
- Health center - student
- Indian Health Service
- Medical practice - family medicine
- Medical practice - pediatrics
- Medical practice - internal medicine
- Medical practice - OB/GYN
- Medical practice - other specialty
- Urgent care

Pharmacy

- Pharmacy - chain
- Pharmacy - independent

Unknown/Other

- Corrections/detention health services*
- Home health care provider
- Long-term care - nursing home, skilled nursing facility, federally certified*
- Long-term care - assisted living*
- Long-term care - intellectural or developmental disability*
- Long-term care - combination (e.g., assisted living and nursing home in same facility)*
- Other
- *no response*

Appendix Figure 1. Provider site categories. * indicates site excluded from analysis.

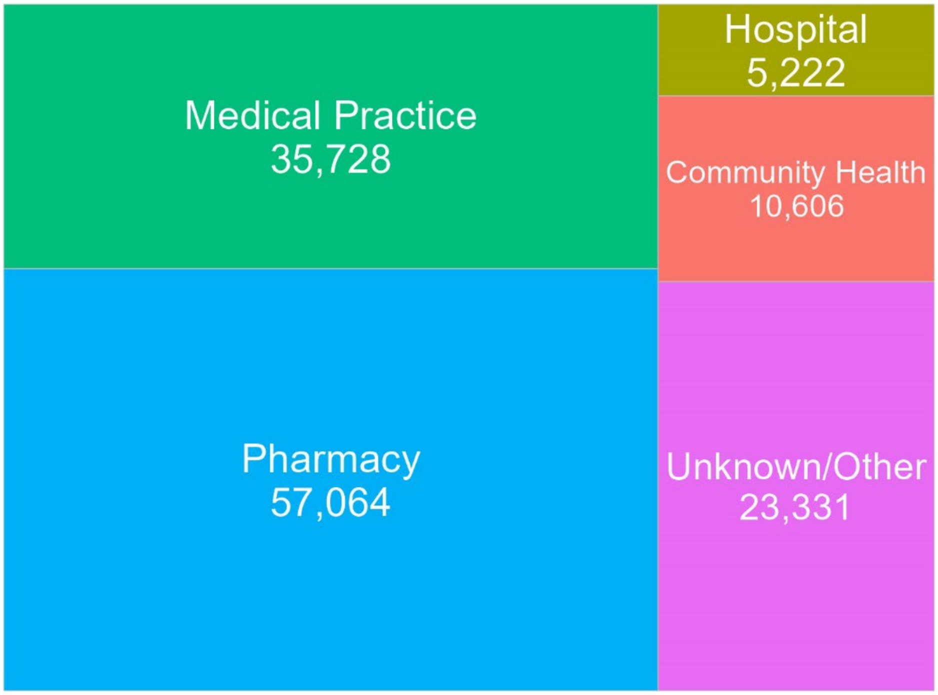

Appendix Figure 2. Total provider sites (n=131,951) by provider category.
